# Unveiling Hidden Prints: Optically stimulated luminescence for latent fingerprint detection

**DOI:** 10.1016/j.heliyon.2023.e22794

**Published:** 2023-11-23

**Authors:** Andrea Pinna, Sofia Rocca, Stefania Porcu, Roberto Cardia, Daniele Chiriu, Carlo M. Carbonaro, Riccardo Corpino, Enrica Tuveri, Pietro Coli, Pier Carlo Ricci

**Affiliations:** a Department of Physics, University of Cagliari, S.p. no. 8 Km 0700, 09042 Monserrato, CA, Italy; b Scientific Investigation Department (RIS) of Cagliari, Piazza San Bartolomeo 29, 09126, Cagliari, CA, Italy

**Keywords:** Optical stimulated luminescence, Applied optics, Fingerprint detection, Ba2SiO4, Forensic applications

## Abstract

Fluorescent lighting and optical techniques have been widely utilized to enhance the detection of latent fingerprints. However, the development of new techniques is imperative to expand the range of surfaces from which latent fingerprints can be detected. When relying on traditional methods, fingerprint evidence can remain undetected or even disregarded due to insufficient detection and limited detail, especially when dealing with a luminescent background.

In this study, we propose the utilization of optically stimulated luminescence (OSL) applied to a Ba_2_SiO_4_ matrix, co-doped with Eu^2+^ and Dy^3+^, as a powerful method for visualizing latent fingerprints on various surfaces, including thin plastic bags, rigid duct tape, thin aluminum foil, and glass slices. This technique effectively eliminates any luminescent background and significantly enhances optical imaging.

This represents the first successful application of OSL in the development of latent fingerprints, thus paving the way for more efficient and effective forensic techniques in the future.

## 1 Introduction

Forensic investigations rely heavily on the detection and development of latent fingerprints. Although traditional techniques for revealing fingerprints on smooth surfaces such as glass or metal have improved over time, researchers are constantly seeking to enhance the process to increase its efficiency and effectiveness [[1], [2], [3], [4], [5]]. The difficulty in detecting and developing latent fingerprints varies depending on multiple factors, including the surface the fingerprint was left on and the elapsed time since it was left. Choosing the appropriate method for each case is crucial, as many techniques are irreversible and using the wrong method can lead to failure in revealing the fingerprint's distinctive features [6]. To overcome this challenge, researchers are investigating the development of materials that can effectively reveal latent fingerprints on surfaces where current techniques fall short [[7], [8], [9]]. These materials need to be versatile enough to be used in various conditions, reducing the number of options available to the fingerprint developer.

Among the available methods, the use of luminescent materials has gained significant popularity thanks to the high efficiency of the process [10,11]. Fluorescent powders are typically applied on latent fingerprints and adhere to the residual organic substances previously left by the finger skin. Thanks to their intense emission, they can enhance fingerprints also in challenging samples where the background can interfere with the trace and reduce the clarity of the ridge pattern for the next analysis phase [3,4,[12], [13], [14]].

However, when the substrate or other substances present near the fingerprints are fluorescent too, it becomes very difficult, and sometimes impossible, to distinguish the fingerprints from them [15].

This problem can be overcome with the use of up-conversion phosphors, as proposed recently [15,16], where a double-photon absorption process causes the emission at lower wavelength than that of the exciting beam, generally in red/infrared region. This phenomenon is suitable for fingerprint detection since it allows to reveal the fingerprints on common fluorescent substrates. However, up-conversion phosphors are intrinsically low efficient and require the use of high intense light sources about 2 W/cm^2^ [16].

A different strategy relies on the use and the control of defects in host matrix to obtain an emission at energy higher than the excitation beam [17,18]. The defects sites in some cases can act as trapping sites for the charges released in the conduction band (electrons) or valence band (hole). When the sample is irradiated with light source of a proper wavelength, the generated electrons and holes can fill these traps, and stored there for a time dependent on the temperature of the sample and depth of the trapping sites. However, if the sample is suitably doped and possesses radiative recombination sites, the charge released upon suitable excitation can generate a radiative emission.

**Fig. 1:**
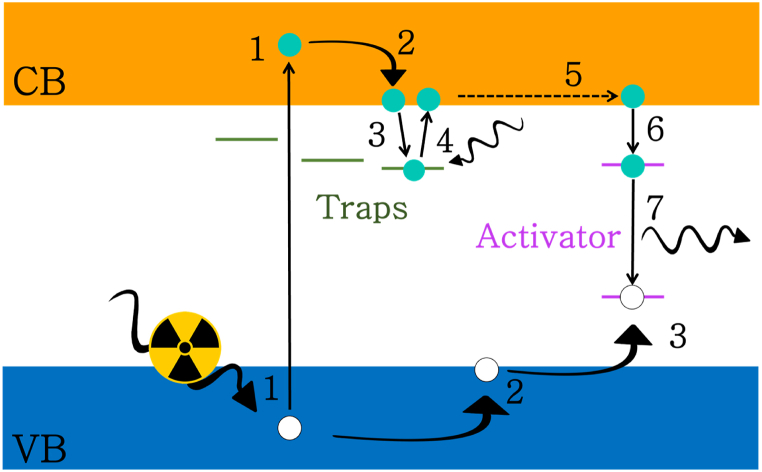
OSL mechanism: (1) irradiation and electron-hole pair generation; (2) charge carriers thermal relaxation; (3) electron trapping in the trapping sites and hole trapping in the activator site; (4) optically stimulated electron excitation in conduction band, (5) electron movement from trapping to activator site; (6) electron trapping in the activator site; (7) electron-hole recombination.

In case of thermal stimulations, the process is called thermally stimulated luminescence [19]; when they are stimulated by photons, the process is known as optically stimulated luminescence (OSL) [17]. OSL is typically performed by stimulating the sample with a red or near-infrared (NIR) light which will typically emit light at lower wavelengths. The exact mechanism of this process can vary in different host-dopant systems and sometimes is far to be trivial [17,18,[20], [21], [22]]. Nevertheless, a simplified and schematic view of this process is shown in Fig. 1, where two different sites between valence band (VB) and conduction band (CB) are present: the trapping and activator sites. Firstly, irradiated by photons of sufficient energy (usually X-rays or UV, but also blue light has been reported), electron-hole pairs are created (1). After thermal relaxations, electron and holes arrive in the bottom and top of CB and VB (2), from which are trapped in different sites (3). Generally, electrons are captured by the trapping sites while holes directly by the activator site. In this condition, the charge-carriers are not able to recombine and can stay trapped for different length of times (from fraction of milliseconds to decades) depending on the energy depth of the trapping sites. Then, where an optical stimulation occurs, the trapped electrons are excited again into the CB (4), where are free to move in the solid (5) until they are either re-trapped or captured by an activator site (5) and finally radiatively recombine with the trapped hole. The wavelength of the emitted photon will depend on the energy levels of the activator and generally in OSL materials, the emission wavelength is lower than the stimulation wavelength. The effect of this process, even lying on a different physical mechanism, is therefore similar to that seen in up-conversion materials but the stimulation can be easily performed also by common and low-cost light emitting diodes (LEDs) and few mw/cm^2^ as stimulation light power density [23,24]. Another great advantage of OSL in latent fingerprint development is that enables to dramatically expand the range of available phosphors, enabling researchers to find the best option in terms of emission efficiency, particle size, chemical composition and surface properties [18].

In this work we have verified for the first time the feasibility to apply the OSL technique for the latent fingerprint detection. Among the different materials already utilized for OSL measurements [17,18], we have optimized and utilized a promising system formed by barium orthosilicate Ba_2_SiO_4_ [25,26], doped with very low amounts of rare earth elements (REEs), and obtained with suitable synthesis conditions. The Ba ionic radius permits an effective doping process with rare earth, further, the synthesis conditions permit to obtain reproducible crystals and optical properties and the OSL process is easily to detect in the visible spectral range. If doped with Eu^2+^ and Dy^3+^, Ba_2_SiO_4_ exhibits an efficient green fluorescence that can be stimulated in OSL, after UV-C irradiation, by infrared/red light stimulation. The co-doping with Dy introduce deep trapping sites in the matrix, while Eu represent efficient radiative channels. The ratio among the rare earth elements is crucial for efficient optical data storage and for OSL measurements and previous detailed studies identified the optical concentration in 0.5mol% for Eu and 0.2mol% for Dy [26].

This marks the first successful application of OSL in the development of latent fingerprints, paving the way for more efficient and effective forensic techniques in the future.

## 2 Materials and methods

### 2.1 Ba_2_SiO_4_ synthesis

The phosphor powders of Ba_2_SiO_4_ were prepared by a conventional solid-state reaction method [26], with a doping concentration of 0.5 mol % of Eu and 0.2 mol % of Dy. The precursors BaCO_3_ (>99 %, Sigma Aldrich, Merck KGaA, Darmstadt, Germany), SiO_2_ (>99.8 %. Supelco, Merck KGaA, Darmstadt, Germany)**,** Eu_2_O_3_ (>99.95 %, Sigma-Aldrich, Burlington, Massachusetts, US) and Dy_2_O_3_ (>99.99 %, Sigma-Aldrich, Burlington, Massachusetts, US) were mixed with 8 % mol of BaF_2_ (>99.95 %, Sigma Aldrich, Merck, Darmstadt, Germany) as a flux. The powder was mixed in an agate mortar and put into an alumina boat and then into a tubular furnace at 1000 °C for 6h. Then the powder was re-grounded and placed in a tubular furnace under reductive 90%Ar/10%H_2_ at 1250 °C for 4h to induce the reduction of Eu^3+^ to Eu^2+^. The annealed powder was re-grounded again, passed through a 20 μm sieve and was finally ready to use.

### 2.2 Fingerprints deposition

The deposition of latent fingerprints was performed following thorough hand washing with water and soap. Subsequently, the donor gently rubbed the forehead with a finger and pressed the finger on the selected substrate for fingerprint development.

Then, the Ba_2_SiO_4_ powder was casted with a fiberglass filament brush (No.122L, Sirchie, Youngsville, North Carolina, US), both with and without the prior application of cyanoacrylate (Cyanobloom, Foster + Freeman, Evesham, UK) before brushing. Cyanoacrylate application was conducted through the common fuming method [27]: the support with the latent fingerprint is suspended in a glass chamber in which two containers with cyanoacrylate and water were placed. The temperature of the chamber is then raised to 120 °C to induce cyanoacrylate fuming. Its vapors then interact with the organic residues on the fingerprints and polymerize in these regions, giving a white appearance to the fingerprint. The water container is necessary to provide sufficient humidity for the reaction to take place efficiently [28].

### 2.3 Photoluminescence and optically stimulated luminescence measurements

An Advantes Sensiline Avaspec-ULS-TEC Spectrometer (spectral range 250–900 nm) was used for PL and OSL measurements. In the first case, the powder was excited with light emitting diode (LED) emitting at 365 nm and the PL signal was acquired by the spectrometer through an optical fiber. The OSL measurements were performed by irradiating the sample with a 655 nm diode laser (10 mW/cm^2^). The excitation was filtered by a shortpass pass filter with a cut-off wavelength of 600 nm.

### 2.4 Thermoluminescence measurements

Thermoluminescence measurements were carried out with a built-in setup. The measurements were performed from 20 °C to 300 °C with a heating ramp set to 10 °C/min. The luminescence was acquired from a phototube working at 1.3 kV (model PM28B, Thorn Emi, London, UK). A home-made software with integrated PID control, regulated the temperature ramp through the connection to power supply (model 6030A, Agilent Technologies, Inc., Santa Clara, California, US) and data acquisition (model 3852A, Hewlett-Packard, Palo Alto, California, US) units.

### 2.5 X-ray diffraction measurements

X-ray measurements were carried out with a Bruker D8 Advance diffractometer operating at 30 kV and 20 mA, equipped with a Cu tube (λ = 1.5418 Å) and a Vantec-1 PSD detector. The measurements were recorded with a zero-background substrate at 2θ ranging from 20° to 90°. Rietveld analysis was carried out with MAUD software [29].

### 2.6 OSL imaging of latent fingerprints

Latent fingerprints were developed by OSL imaging using a red-light emitting diode (LED) for optical stimulation (10 mW/cm^2^) and a shortpass filter with a cutoff at 600 nm for blocking the diffused LED light. The transmitted OSL signal was acquired by the digital camera of a commercial smartphone (Samsung S10E, Samsung Electronics Co., Ltd., exposure time: 30s).

## 3 Results and discussion

**Fig. 2:**
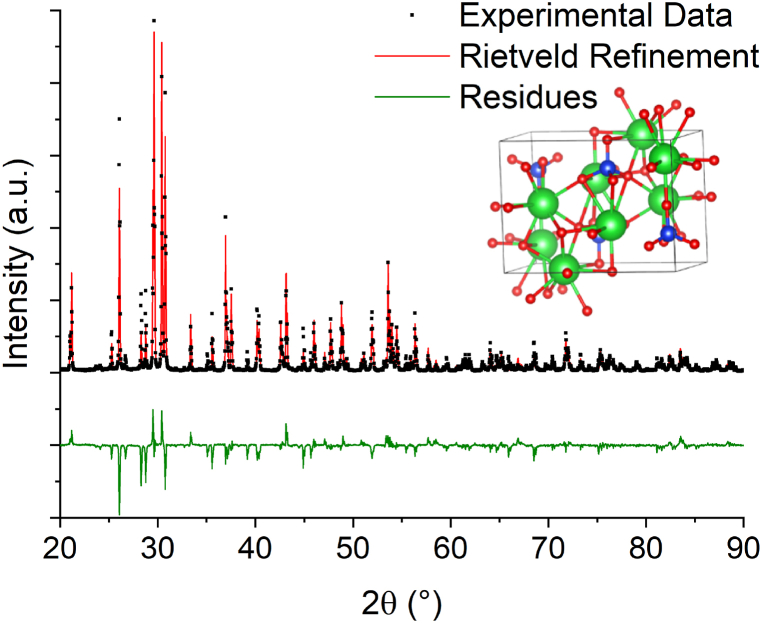
XRD pattern of Ba_2_SiO_4_ powder. In the inset: crystal structure of the identified orthorhombic phase.

Fig. 2 displays the XRD pattern of the synthesized Ba_2_SiO_4_ powders co-doped with Eu and Dy. The Rietveld refinement confirms the successful production of barium orthosilicate through solid-state reaction with a purity of 98 mol.%. Some minor peaks are attributed to non-reacted BaF_2_ (Space group Fm-3m) with a concentration of 0.5 mol.%, and to Barium dialuminate (Space Group P63) with a concentration of 1.5 mo%. The presence of such phase can be caused by the diffusion of Al atoms from the alumina boats in which the synthesis was carried out. The uniformity of the crystal structure pays a very high importance on the optical properties and in particular on the OSL properties. The presence of secondary phase can generate non radiative channels in the border of the different phase, strongly decreasing the light emitted.

Ba₂SiO₄ crystallizes in the orthorhombic *Pnma* space group. There are two inequivalent Ba^2^⁺ sites that are the substitutional sites for the rare earth elements doping. Ba^2^⁺ is bonded in a 9-coordinate geometry to nine O^2^⁻ atoms and in a 10-coordinate geometry to ten O^2^⁻ atoms, in the first and second sites respectively. In the first site the Ba–O bond distances spread between 2.70 and 3.13 Å while in the second Ba^2^⁺ site, the Ba–O bond distances ranging from 2.66 to 3.22 Å. Si⁴⁺ is bonded in a tetrahedral geometry to four O^2^⁻ atoms with Si–O bond distances between 1.63 and 1.65 Å. Oxygen possesses three inequivalent O^2^⁻ sites. The cell parameters calculated by Retvield refinement are the following: a: 5.810 Å, b: 10.218 Å, c: 7.507 Å.

The Raman spectrum (Fig. S1) confirms the crystalline structure of the matrix and no extra band from secondary phases have been observed. The most intense modes at 381, 505, 530, 831, 867, 897, 919 cm^−1^ are assigned to the characteristic vibrational modes of the tetrahedral group (SiO_4_)^4−^, where the most intense peak (831 cm^−1^) can be attributed to the symmetric stretching of the tetrahedron. As expected, the XRD and Raman measurements didn't show any appreciable change related to the different dopant compositions, due to their very low concentrations.

**Fig. 3:**
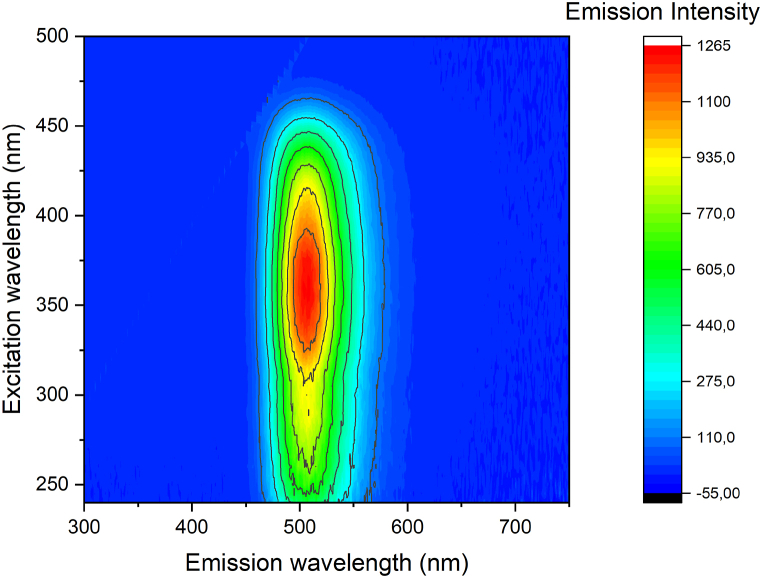
PLE map of Ba_2_SiO_4_:Eu,Dy sample.

Fig. 3 shows, in a 3D plot (excitation wavelength, emission wavelength, emission intensity), the emission and excitation properties of the sample. The broad emission between 450 and 550 nm possesses a very wide excitation spectral range, that spans from the deep UV (250 nm) to visible 470 nm. The main excitation path, anyway, lies in the near UV region between 350 and 390 nm that involves the direct excitation of the Eu level [30,31] and permits to obtain a high internal quantum yield (IQY), around 71 % (Fig. S2). As expected, due to the trapping nature of the Dy^3+^ ions in the matrix, the co-doping Eu–Dy slightly decreases the IQY to 66 %, however, since the excitation is resonant with the Eu^2+^ channels, the effect is relatively low and the time decay of the fluorescence behavior monitored at 500 nm confirms that there is no significant charge transfer processes from Eu to Dy (Fig. S3). The presence of a charge transfer process would induce a shortening of the time decay constant obtained from time resolved measurements, due to the increased probability of non-radiative channel. The efficiency of the transfer is [32,33]:

$$\sigma =1-\frac{\tau_{s}}{\tau_{0}}$$

Where τ_s_ and τ_0_ indicate the time decay constant of Eu^2+^ the with and without the Dy^3+^. Considering the time resolved measurements and the fitting procedure (Fig. S3), the efficiency is almost negligible (τ_s_ = 635 μs and τ_0_ = 655 μs, σ = 0.03).

It is well known that Europium acts as recombination channel, where in the 2+ oxidation state involves the ^4^f_7_(^8^S_7/2_) → ^4^f_6_^5^d_1_ (hereafter 4f → 5d) levels. Further, unlike to 3+ charge state of rare earth elements, the electronics of 5d levels are exposed to the external electronic shell therefore the optical properties are strongly affected by the surrounding environment of Eu^2+^ ions, such as crystal symmetry, atom coordination, and crystal field strength, generating the broad emission [30]. On the contrary, the emissions from Dysprosium ions (Dy^3+^) are relatively narrow and located at 480, 575 and 680 nm regardless the host matrix. In our sample, we did not observe any emissions from Dy^3+^, suggesting that its primary role is related to the defects formation rather than acting as an emitting center.

**Fig. 4:**
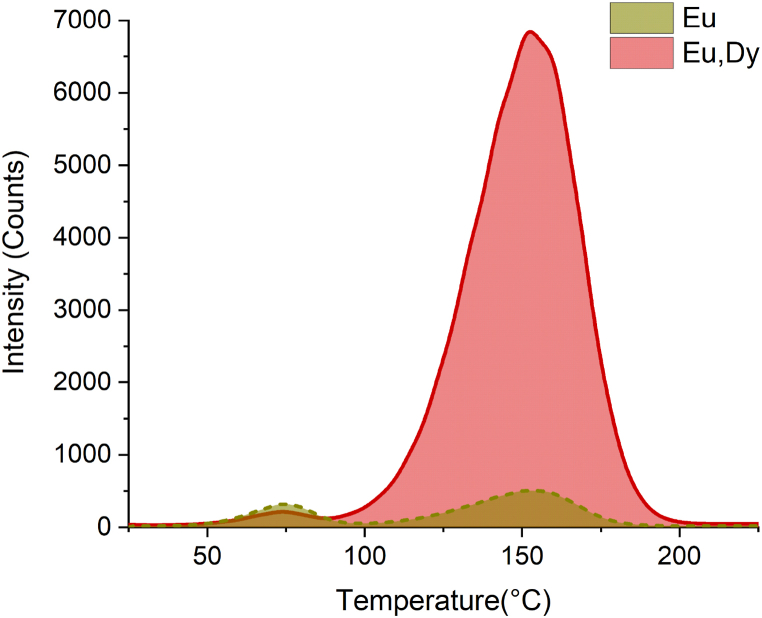
Thermoluminescence of Ba_2_SiO_4_:Eu and Ba_2_SiO_4_:Eu,Dy sample.

The presence of trapping center is well underlined by the thermoluminescence measurements (Fig. 4). The samples were irradiated with band-to-band excitation at 254 nm and subsequently heated at 10 °C/min. The peak maximum is observed at 160 °C for all the samples, but in the co-doped sample (Dy^3+^, Eu2^+^) the emission is strongly increased (about two orders of magnitude for the same amount of powder with respect the single Eu^2+^ sample acquired with the same experimental conditions), suggesting that the trapping site is connected to the presence of the co-dopant but that it is still present in the matrix itself. As an example, it can be due to the formation of Oxygen vacancies generated during the co-doping process to compensate the charge balance Ba^2+^→Dy^3+^. However, the nature of such defect site is still to be discussed and further dedicated measurements and analysis are mandatory to better define it, but it is out of the scope of the present work.

**Fig. 5:**
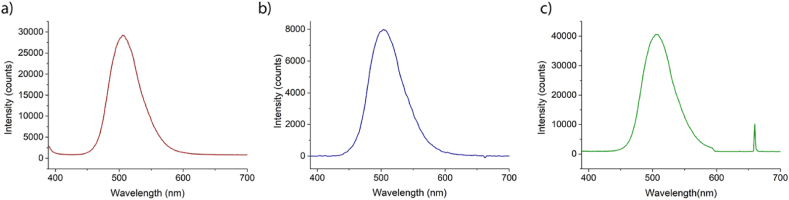
Emission spectra of Ba_2_SiO_4_ powders in a) photoluminescence (excitation wavelength: 365 nm), b) thermoluminescence at 150 °C, c) optically stimulated luminescence.

Fig. 5a showcases the photoluminescence of the phosphor, Ba_2_SiO_4_:Eu,Dy, upon excitation by a 365 nm emitting LED. The emission displays a distinctive broad band related to the Eu^2+^ 5d-4f transition, centered at around 505 nm. The spectral dependance does not change if excited at 254 nm as indicated in Fig. 3. Fig. 5b shows the spectral resolved emission of the thermoluminescence curve, acquired at 150 °C. As it can be easily observed there is no variation, indicating once more, that emitting centers involve the Eu^2+^ levels.

Finally, the optically stimulated luminescence (OSL) was also observed, (Fig. 5c): the powder previously excited at 254 nm (3 min, optical flux 0.5 mW/cm^2^), were illuminated at 655 nm (1 mW/cm^2^) and the emission was monitored with a short wavelength filter at 600 nm. Again, the emitting center involved comes from the Eu^2+^ levels.

It is worth to note that the thermoluminescence does not give any signal after the excitation at 655 nm, indicating that the defects sites involved are the same.

The SEM image reported in Fig. S4 shows the details of the powder obtained, as well, the EDS analysis confirm the stoichiometric composition of the sample (Table S1). The powders show an average size between 10 and 20 μm. Smaller powder sizes are associated with better latent fingerprint development since adhesion is enhanced [34]. However, OSL efficiency is known to decrease with particle size, as it heavily depends on crystalline order [35,36]. Moreover, particle size also affects the safety of operators. Particles with sizes less than 10 μm (PM10) and especially less than 2.5 μm (PM2.5) are considered dangerous when inhaled, causing severe health problems and exposing the operator to risks. For these reasons, we did not perform further treatments to decrease the particle size any further.

**Fig. 6:**
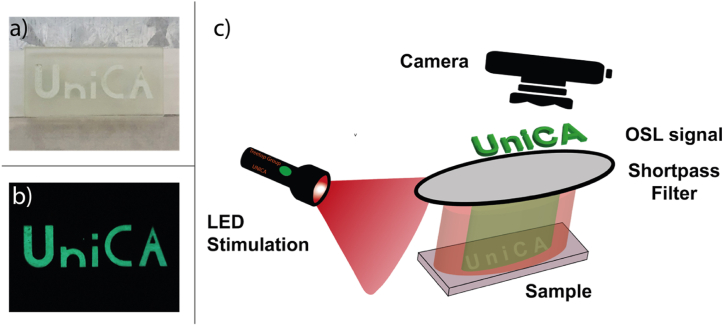
a) photograph under room lighting and b) OSL imaging of the of a sample with Ba2SiO4 powders. c) OSL imaging setup composed by a LED source of optical stimulation, the sample, a shortpass filter for reflecting the diffused LED light and a camera for collecting the transmitted OSL signal.

The efficiency of the optically stimulated process (OSL) is depicted in Fig. 6, where the image obtained from the OSL emission was captured using the camera of a standard mobile device with the experimental setup described in Panel C. The powder was irradiated with UV light and subsequently deposited on a glassy transparent support with the word 'UniCA' (short for 10.13039/100013003University of Cagliari) (Fig. 6, Panel a). The OSL image (Fig. 6, Panel b) was acquired by illuminating it with a red light (LED at 650 nm - 10 mW/cm^2^) through a short-pass filter (at 600 nm)."

**Fig. 7:**
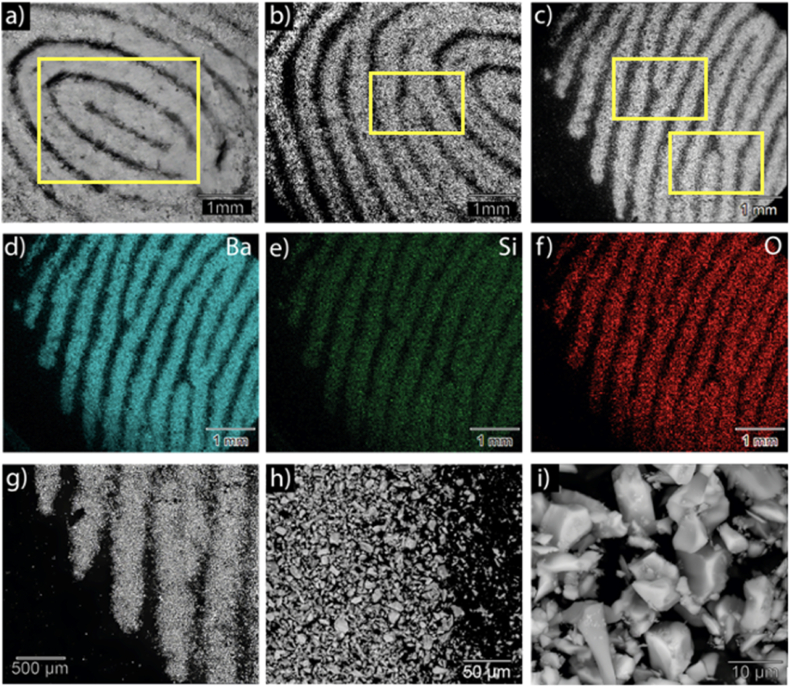
a), b), c): SEM images of different areas of a latent fingerprint developed with Ba_2_SiO_4_ powders; d), e), f): EDS maps of Ba, Si and O elements carried out in the same area of image c); g), h), i) SEM image of the latent fingerprint details at increasing magnification levels.

Fig. 7 shows the fingerprint deposited on an aluminum substrate and observed through SEM analysis. It is clearly visible that the powder adheres only on the fingerprint residues and well reproduce the typical arches, loops and whorls. The EDX analysis as well the enlarged view, give further details on the deposited powder.

The powder was thoroughly studied to determine the best method for developing fingerprints. It was applied in two different ways: immediately after deposition and after 48 h. The application was done directly to the latent fingerprint or after undergoing cyanoacrylate fuming, a typical process used for revealing latent fingerprints by deposition of cyanoacrylate vapor to the substances left by the skin. This process is commonly used in the forensic scene as it efficiently binds to fingerprint residues even after years of deposition. Due to its popularity, it is crucial that a newly developed powder for latent fingerprints also works on cases where cyanoacrylate fuming is applied.

**Fig. 8:**
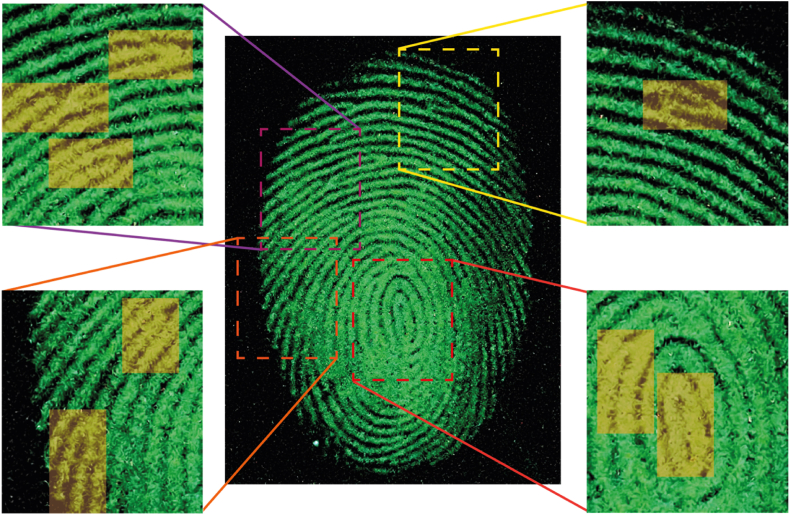
OSL fingerprint imaging. The insets show the characteristic details of the fingerprint.

The OSL image (Fig. 8) of the same fingerprint as shown in Fig. 7 was acquired after pre-exciting the powder with UV and using the same experimental apparatus as depicted in Fig. 6c. Notably, the insets in the figure clearly and distinctly display the unique details of the fingerprint. These distinguishing features are essential in facilitating the accurate identification of the individual who left the fingerprint, making the utilization of OSL imaging highly relevant and valuable in forensic investigations.

In addition to the observations reported in Fig. 8, further study was conducted on the development of latent fingerprints on aluminum substrates by casting the silicate powder after 48 h from fingerprint deposition (Fig. S6). The fingerprints were precisely captured after 48 h. These results demonstrate that aluminum substrates offer an effective surface for latent fingerprint imaging without the need for additional steps.

The feasibility of this method was further tested on different substrates. Figs. S7a and S7b display the OSL image of fingerprints on glass slide immediately after deposition and after 72 h without any additional treatment, while Fig. S7c displays a fingerprint developed on the same substrate after cyanoacrylate fuming. The distinct ridge and valley patterns of the fingerprints are clearly visible, demonstrating the effectiveness of OSL imaging in developing latent fingerprints. The use of cyanoacrylate fuming prior to powder brushing also enhances the preferential adhesion of the powder to the fingerprint ridges, resulting in a clearer and more distinct image.

**Fig. 9:**
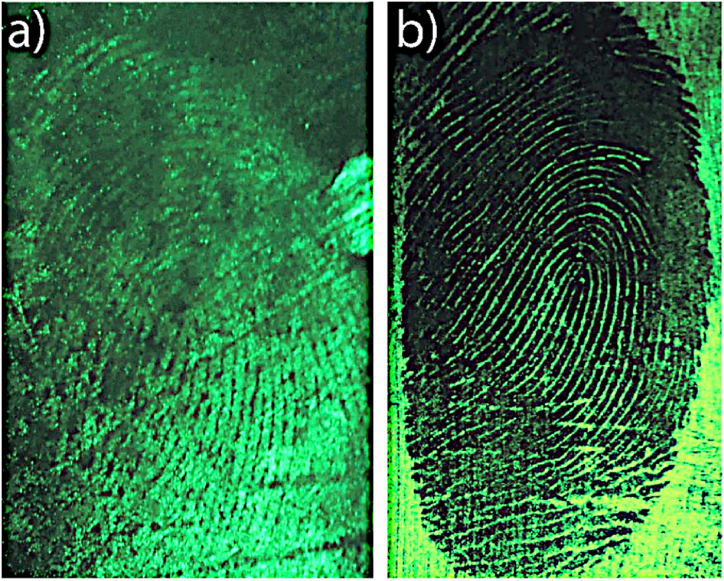
OSL fingerprint imaging on a plastic bag after 48 h from fingerprint deposition (a)) and on a plastic tape right after fingerprint deposition and cyanoacrylate fuming.

Fig. 9 reports the OSL imaging obtained on a plastic bag after 48 h from the deposition and on a plastic tape after the cyanoacrylate treatment. In this last case it is interesting to note that due to the adhesive surface, the powder adheres in all the region apart the zone where the cyanoacrylate itself adhere. The result is a “negative OSL image” between the image captured without the cyanoacrylate treatment (as can be verified from the external zone to the fingerprint).

**Fig. 10:**
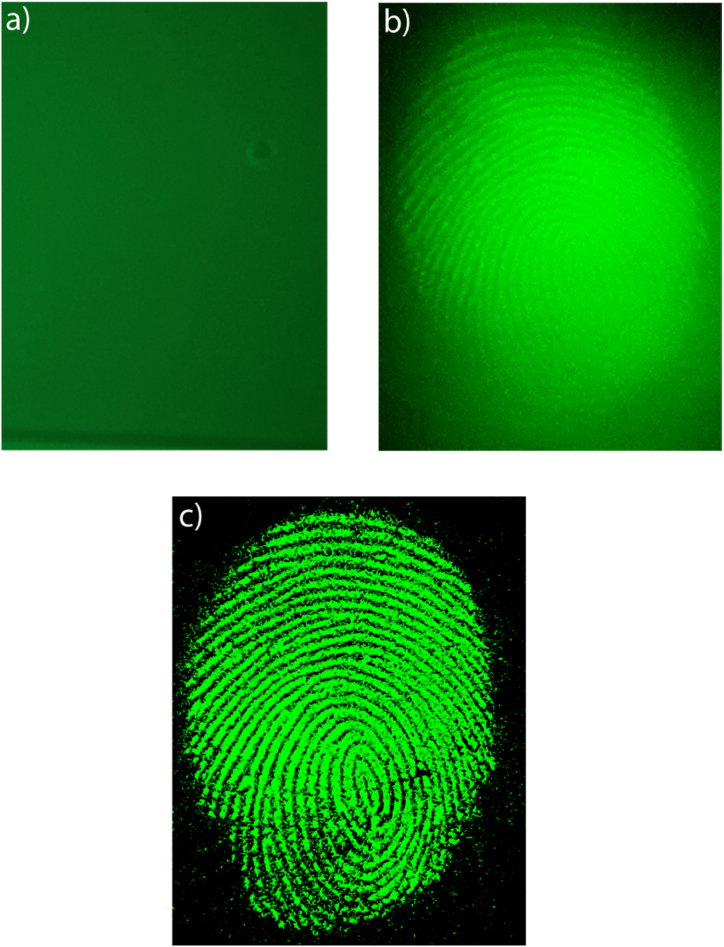
a) fluorescence imaging of latent fingerprint on glass above coumarine solution, b) fluorescence and c) optically stimulated luminescence imaging of latent fingerprint developed with Ba_2_SiO_4_ powder.

To further showcase the practical application of this method and to evidence the advantage of the OSL imaging, we performed a comparison with the traditional fluorescence imaging for latent fingerprint detection. First, we created a fluorescent glass slice by immersing it in a Coumarin 481 fluorescent substance, and then we imprinted a fingerprint on it, which is not identifiable by optical methods and fluorescent imaging (see Fig. 10a). Subsequently, we applied Ba2SiO4 powders to the fingerprint, capturing both traditional fluorescence images (the sample has a quantum yield (QYI) of more than 0.70) and OSL images (as shown in Fig. 10b).

While the fluorescence image displayed some fingerprint details, they were challenging to distinguish due to the background fluorescence. In contrast, the OSL image (Fig. 10c) provides a much clearer view of the fingerprint details, effectively overcoming the background fluorescence. The data and imaging reported in this study underscore the remarkable potential of Optically Stimulated Luminescence (OSL) in the realm of forensic applications. Not only does OSL offer the promise of enhancing the efficiency and accuracy of latent fingerprint detection, but it also presents a groundbreaking opportunity for advancing the state of forensic science, providing valuable insights that may lead to more effective crime scene investigations.

## 4 Conclusions

There is a pressing need for the development of new techniques to further expand the range of surfaces from which latent fingerprints can be effectively detected. Traditional methods often fall short in detecting fingerprint evidence, leading to potential oversight or dismissal, particularly in cases involving a luminescent background.

The results showed the potential of this method in developing clear and concise fingerprints.

This study proposes a powerful method for visualizing latent fingerprints by employing optically stimulated luminescence (OSL) on a Ba_2_SiO_4_ matrix co-doped with Eu^2+^ and Dy^3+^. The application of OSL successfully overcomes the challenges posed by luminescent backgrounds and greatly improves optical imaging. The versatility of this technique is demonstrated through its successful detection of latent fingerprints on various surfaces, including thin plastic bags, rigid duct tape, thin aluminum foil, and glass slices. This research marks the first successful application of OSL in the development of latent fingerprints, representing a significant milestone in forensic science. The findings pave the way for the future development and implementation of more efficient and effective forensic techniques. With continued advancements in OSL and related technologies, latent fingerprint detection can be enhanced, contributing to more accurate and comprehensive investigations in the field of forensic analysis.

## Data availability statement

Data will be made available on request.

## CRediT authorship contribution statement

**Andrea Pinna:** Conceptualization, Formal analysis, Writing – original draft, Investigation. **Sofia Rocca:** Formal analysis, Investigation. **Stefania Porcu:** Formal analysis, Investigation. **Roberto Cardia:** Investigation, Validation. **Daniele Chiriu:** Formal analysis, Investigation. **Carlo M. Carbonaro:** Investigation. **Riccardo Corpino:** Investigation. **Enrica Tuveri:** Formal analysis, Investigation, Supervision. **Pietro Coli:** Formal analysis, Visualization. **Pier Carlo Ricci:** Conceptualization, Formal analysis, Investigation, Supervision, Writing – original draft, Writing – review & editing.

## Declaration of competing interest

The authors declare that they have no known competing financial interests or personal relationships that could have appeared to influence the work reported in this paper.
