# Supplementary material for: Unveiling Hidden Prints: Optically stimulated luminescence for latent fingerprint detection
Source: Heliyon. 2023 Nov 23;9(12):e22794. doi: 10.1016/j.heliyon.2023.e22794 (PMC10696211; doi:10.1016/j.heliyon.2023.e22794)
Supplement: Multimedia component 1 [file mmc1.docx]

# Supporting Information for

# *Unveiling Hidden Prints: Optically Stimulated Luminescence for Latent Fingerprint Detection*

# Andrea Pinna ^a^, Sofia Rocca ^a^, Stefania Porcu ^a^, Roberto Cardia ^b^, *Enrica* Tuveri ^b^, Daniele Chiriu ^a^, Carlo M. Carbonaro ^a^, Riccardo Corpino ^a^, Pietro Coli ^b^, Pier Carlo Ricci ^a^*

# ^a^ Department of Physics, University of Cagliari, S.p. no. 8 Km 0700, 09042 Monserrato, CA, Italy;

^b^ Scientific Investigation Department (RIS) of Cagliari, Piazza San Bartolomeo 29, 09126 Cagliari CA (Italy);

* Corresponding author: carlo.ricci@dsf.unica.it


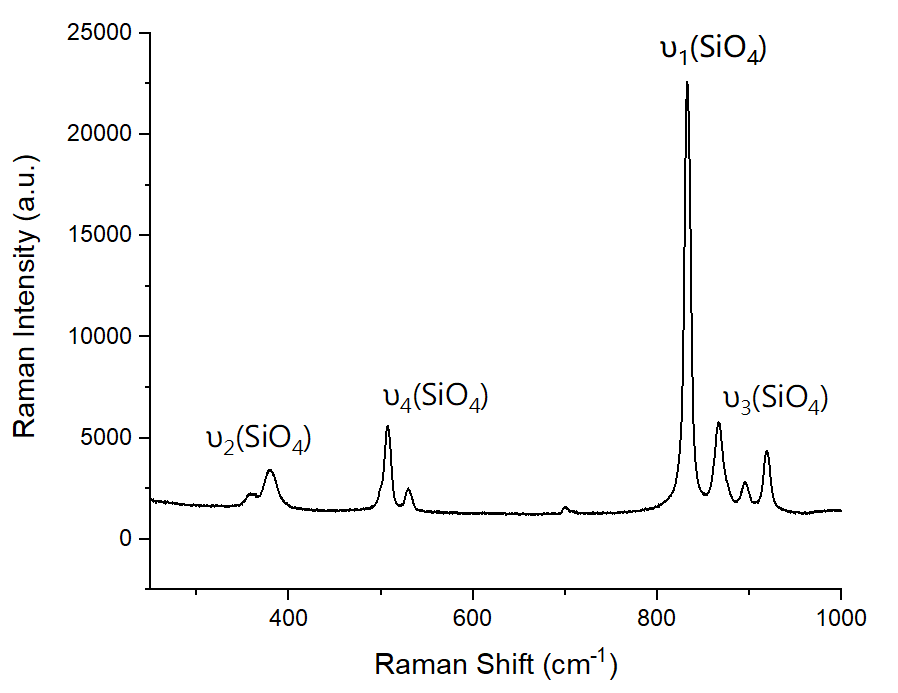


Figure S1 Raman spectrum of Ba_2_SiO_4_ powder.


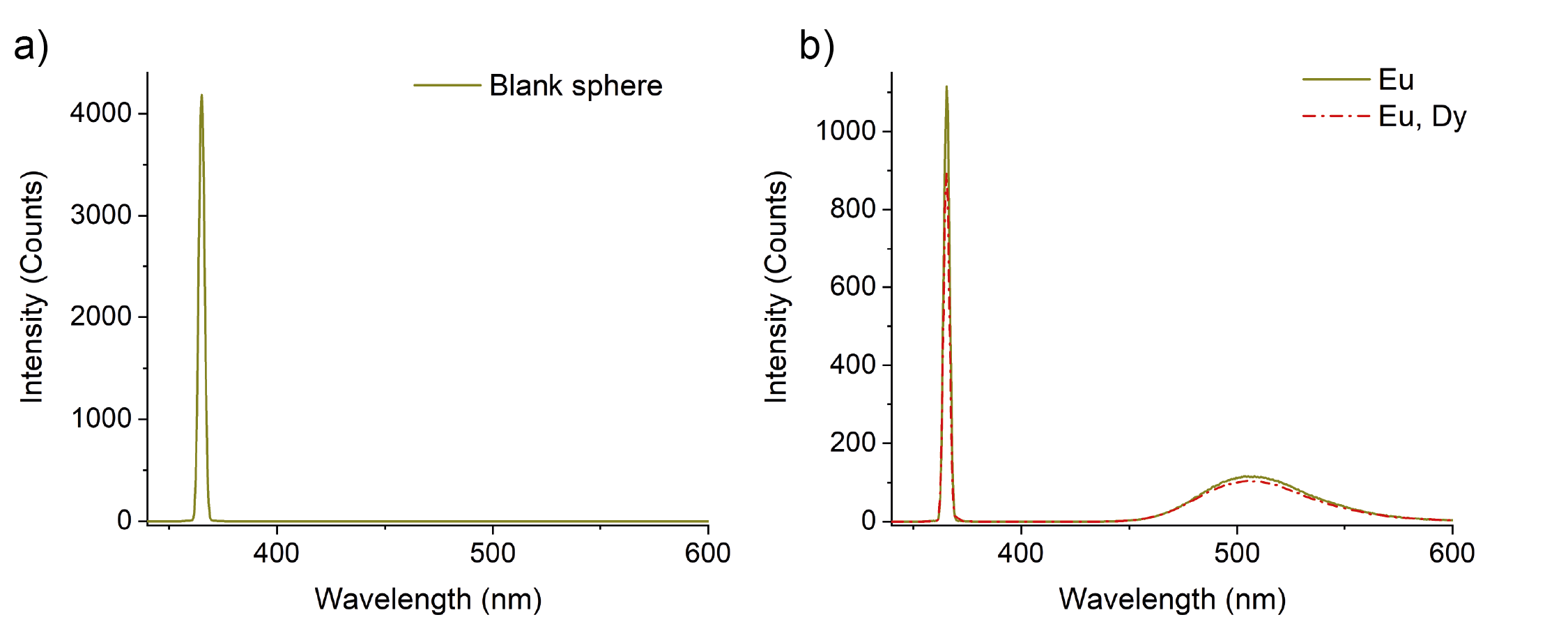


Figure S2: Excitation and emission measurements for quantum yield calculation of (a) the integrating sphere without any sample and of (b) Ba_2_SiO_4_:Eu^2+^ without and with Dy3+ as co-dopant.

IQY was calculated as the ratio between total number of emitted photons ${\#}_{em}$ and the absorbed photons ${\#}_{a}$, i.e. the difference between the number of excitation photons revealed in the blank sphere ${\#}_{ex\_b}$and the number of the excitation photons revealed when the sample was inserted ${\#}_{ex\_s}$:

$$IQY=\frac{{\#}_{em}}{{\#}_{a}}=\frac{{\#}_{em}}{{\#}_{ex\_b}-{\#}_{ex\_s}}$$

*
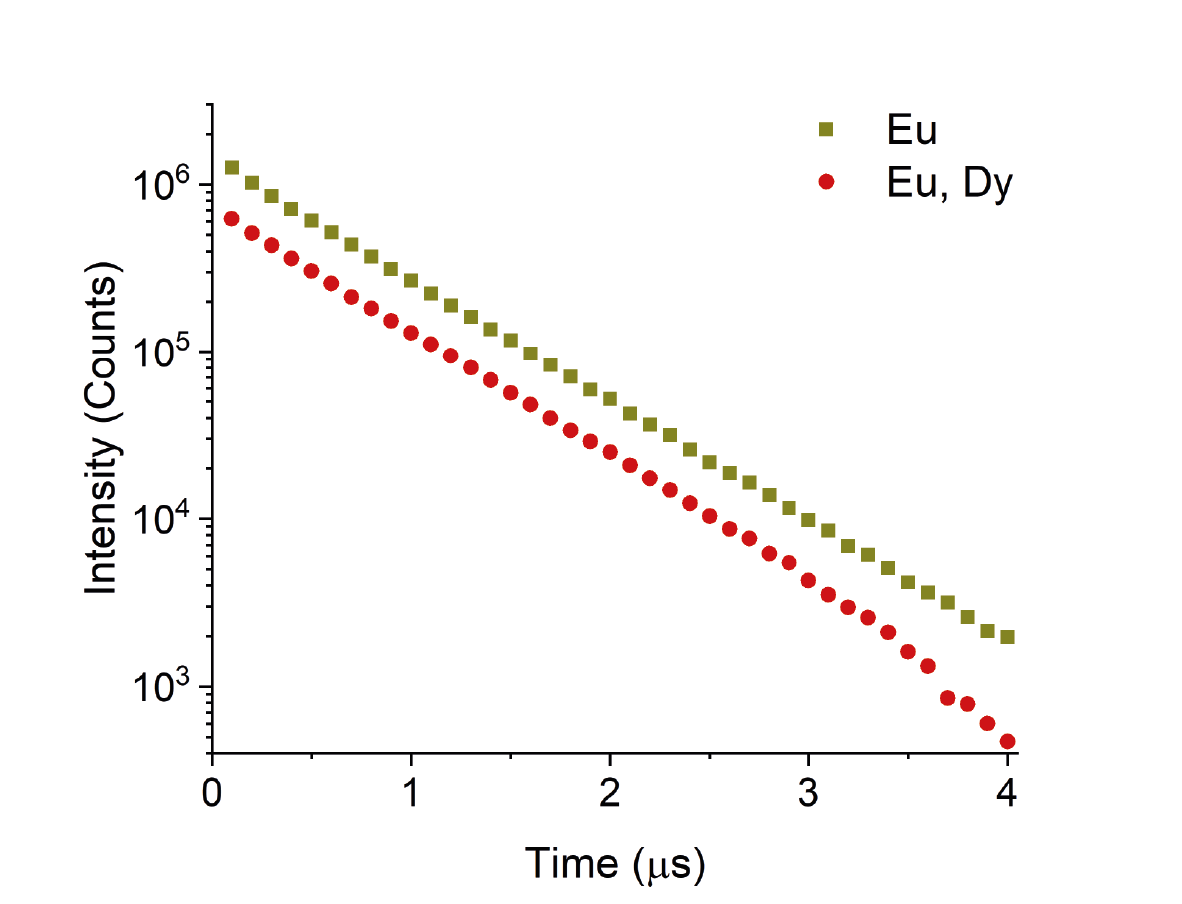
*

Figure S3: Decay time of Ba_2_SiO_4_:Eu2+ without and with Dy3+ as co-dopant.


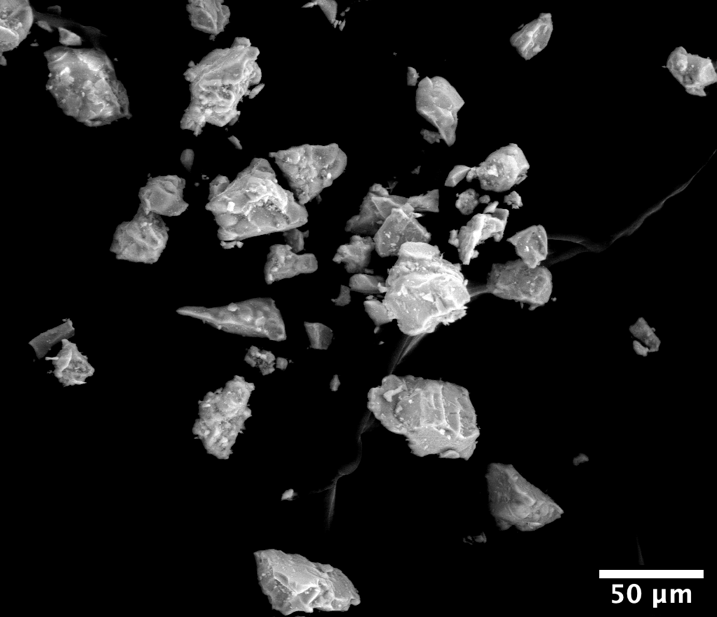


Figure S4 SEM images of Ba2SiO4 powders used for latent fingerprint development.


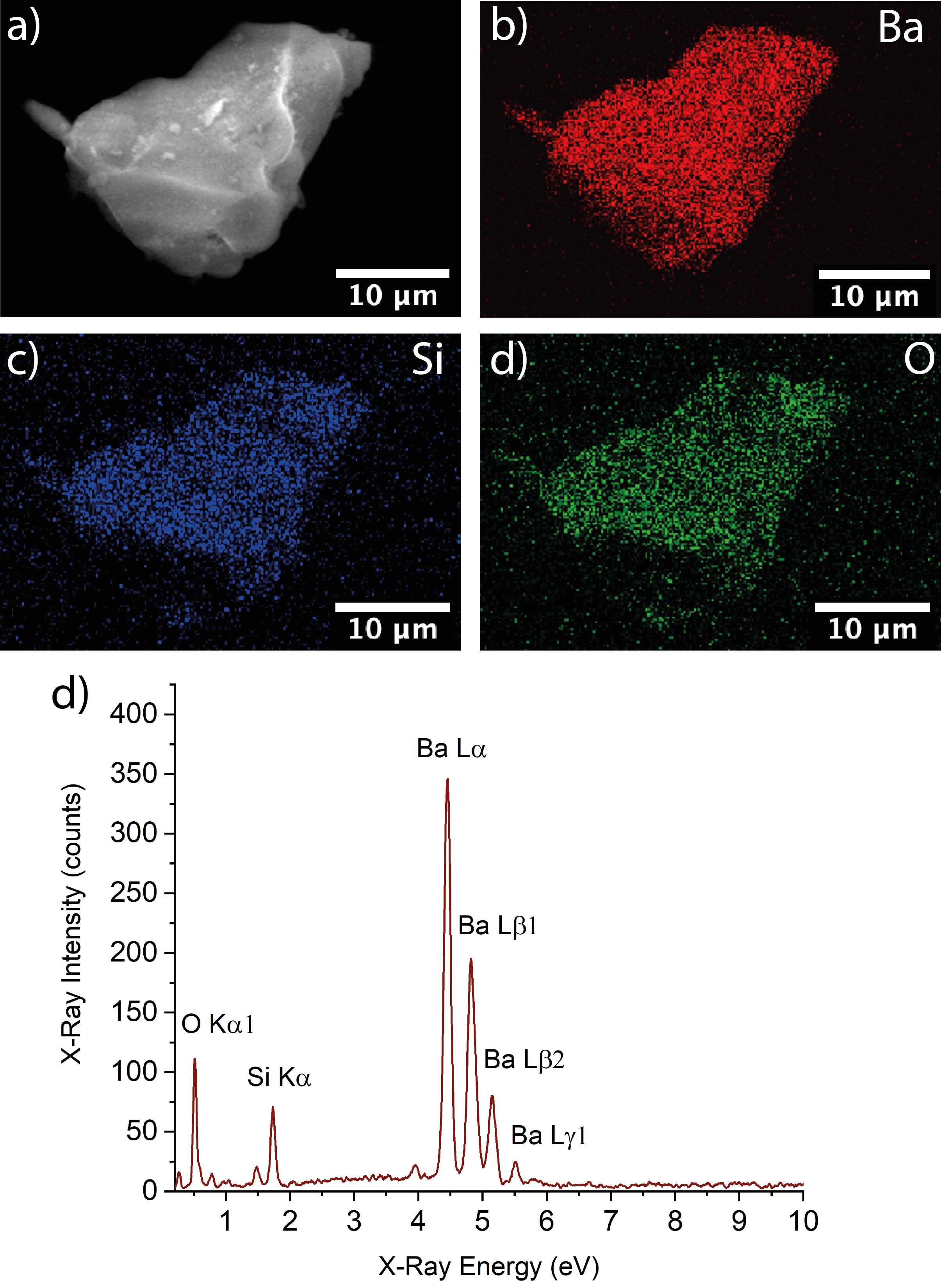


Figure S5 a) SEM image of a Ba2SiO4 powder after sieving with a 20 µm sieve and EDS maps of Ba (b)), Si (c)), O (d)) elements. E) EDS spectrum of the powder.

| ELEMENT |  | | | |
| --- | --- | --- | --- | --- |
|  | Fluorescence Line | Weight % | Mol % | Mol % Error |
| O K | K | 17.6 | 57.8 | 2.4 |
| Si K | K | 7.1 | 13.3 | 0.7 |
| Ba L | L | 75.3 | 28.9 | 0.6 |
|  |  | 100.0 | 100.0 |  |

Table S1 Concentration of O, Si, Ba elements calculated from EDS measurements on Ba_2_SiO_4_ powder.


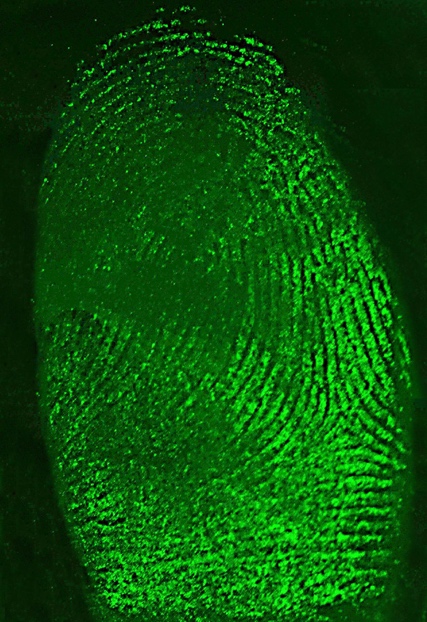


Figure S6 Fingerprint developed after 48 h from deposition on an aluminium substrate.


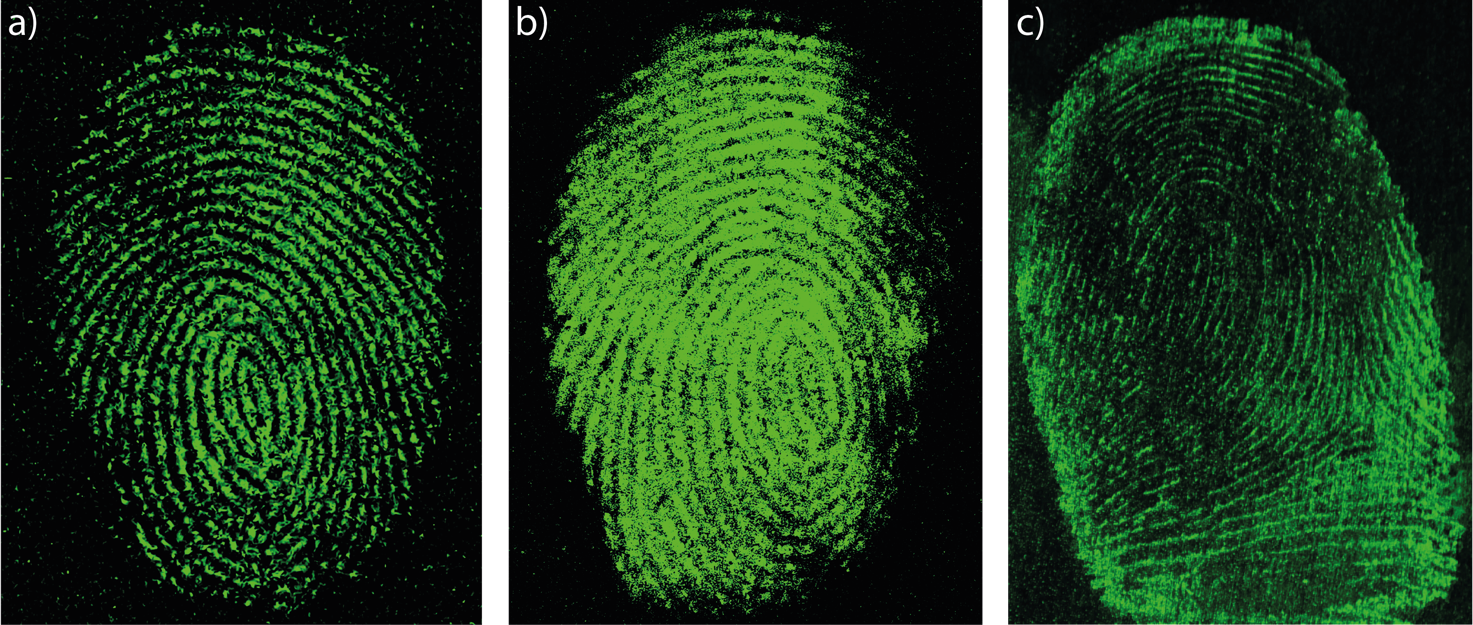


Figures S7a, S7b and S7c OSL imaging of Ba2SiO4 powders casted on microscope glass slides righ after fingerprint deposition (a)), 72 h after the deposition (b)) and after fingerprint deposition followed by cyanoacrylate fuming (c))
